# SARS-2 COVID-19-induced immunity response, a new prognostic marker for the pregnant population correlates inversely with neonatal Apgar score

**DOI:** 10.1007/s15010-022-01773-3

**Published:** 2022-03-05

**Authors:** M. Marwah, H. Shokr, A. Demitry, K. Wang, S. Ahmad, S. Marwah, F. Wandroo

**Affiliations:** 1 Aston Medical School, Aston University, Gosta Green, Birmingham, B4 7ET UK; 2 Pharmacy Division, School of Health Sciences, University of Manchester, Oxford Street, Manchester, M13 9PL UK; 3 Department of Obstetrics, Sandwell and West, Birmingham Hospitals NHS Trust, Hallam Street, West Bromwich, B71 4HJ UK; 4 Department of Haematology, Sandwell and West, Birmingham Hospitals NHS Trust, Hallam Street, West Bromwich, B71 4HJ UK

**Keywords:** COVID-19, Pregnancy, Biomarkers, Perinatal health

## Abstract

**Background:**

The COVID-19 infection has impacted pregnancy outcomes; however, few studies have assessed the association between haematological parameters and virus-related pregnancy and neonatal outcomes. We hypothesised differences in routine haematology indices in pregnant and non-pregnant COVID-19 patients as well as COVID-19-negative pregnant subjects and observed neonatal outcomes in all pregnant populations. Further, we tested if pattern identification in the COVID-19 pregnant population would facilitate prediction of neonates with a poor Apgar score.

**Methods:**

We tested our hypothesis in 327 patients (111 COVID-19-positive pregnant females, 169 COVID-19-negative pregnant females and 47 COVID-19-positive non-pregnant females) in whom standard routine laboratory indices were collected on admission.

**Results:**

Pregnant COVID-19-positive patients exhibited higher WBC, neutrophil, monocyte counts as well as neutrophil/lymphocyte and neutrophil/eosinophil ratio compared to non-pregnant COVID-19-positive patients (*p* = 0.00001, *p* = 0.0023, *p* = 0.00002, *p* = 0.0402, *p* = 0.0161, *p* = 0.0352, respectively). Preterm delivery was more prevalent in COVID-19-positive pregnant patients accompanied with a significantly lower birth weight (2894.37 (± 67.50) g compared with 3194.16 (± 50.61) g, *p* = 0.02) in COVID-19-negative pregnant patients. The COVID-19-Induced Immunity Response (CIIR) was defined as (WBC × neutrophil) / eosinophil; Apgar scores were significantly and inversely correlated with the CIIR index (r =—0.162).

**Interpretation:**

Pregnancy appears to give rise to an increased immune response to COVID-19 which appears to protect the mother, however may give rise to complications during labour as well as neonatal concerns. CIIR is a simple metric that predicts neonatal distress to aid clinicians in determining the prognosis of COVID-19 and help provide early intensive intervention to reduce complications.

## Introduction

Severe Acute Respiratory Syndrome Coronavirus-2 (SARS-CoV-2) is a ribonucleic acid (RNA) respiratory virus responsible for the Coronavirus disease 2019 (COVID-19) pandemic. COVID-19 presentation ranges from asymptomatic, through to mild influenza-like symptoms to multiple organ failure and death [1, 2]. The rapid spread of the virus overwhelmed healthcare systems worldwide, consequently vulnerable and high-risk populations including pregnant women were identified to optimise their management [3]. Pregnant women are particularly prone to respiratory pathogens, like SARS-CoV-2, owing to physiological changes during pregnancy such as increased oxygen requirements and diaphragm elevation making pregnant women susceptible to hypoxia [4].

Pregnant women are considered one of the most unique groups owing to the infection affecting the mothers and their neonates. There is accumulating evidence on pregnant women with COVID-19 suggesting whilst pregnant women are not at an increased risk of morbidity, there is an increased risk for intensive care provision, need for intubation and neonatal distress [5–7]. Studies suggest risk of acute infections is higher in the late stages of pregnancy [8, 9] with an increased incidence of caesarean delivery due to obstetric indications. Furthermore, foetal distress has also been noted in several studies [6, 10, 11].

Hospitals across the world have been collecting data prospectively since patients with COVID-19 first presented, looking for patterns in clinical findings and routine laboratory markers that may predict risk of a poor health outcome in a variety of patient groups. Haematological blood parameters and indices including differential white blood cell count, plasma platelets concentration, platelets and red blood cells distribution width, neutrophil/lymphocyte ratio (NLR), neutrophil/basophil ratio (NBR), neutrophils/ eosinophils (NER) ratio are part of the well-established parameters of inflammatory responses used as simple and reliable prognostic indicators used guide interventions [12–15]. A meta-analysis observed COVID-19-positive pregnant patients to have elevated neutrophils (71.4%; 95% CI 38.5–90.9), elevated CRP (67.7%; 95% 50.6–81.1), and low haemoglobin (57.3%; 95% CI 26.0–87.8) as well as a preterm birth rate of 34.2%, and caesarean section rate of 82.7% [16]. However, few studies have assessed the association between the haematological parameters in COVID-19-positive pregnant patients and virus-related pregnancy and neonatal outcomes [17].

This study aimed to analyse the haematological laboratory parameters, clinical manifestations, maternal and perinatal outcomes in COVID-19 infected pregnant women and compare with the non-pregnant population of reproductive age. We hypothesised that certain routine haematological indices may be altered in those pregnant patients with COVID-19 in whom neonates may consequently be adversely affected, and that pattern identification would facilitate prediction of those at high risk of severe disease and foetal distress.

## Methods

### Study design and participants

This retrospective cohort study included all women (pregnant and not pregnant) with a confirmed diagnosis of COVID-19 as well as a cohort of randomly selected group of pregnant women without COVID-19 infection admitted between 3rd of February 2020 and the 31st of March 2021 at a single centre National Health Trust in the UK.

Data from 380 participants were initially screened for study inclusion, of which 53 individuals were excluded due to incomplete of plasma biomarkers analysis. All patient results were collected within 3 days of COVID-19 diagnosis and, for COVID-19-negative participants, samples were collected during pregnancy booking bloods (less than 10 weeks and again at 24 weeks) following the UK antenatal care national guidelines [18].

**Fig. 1:**
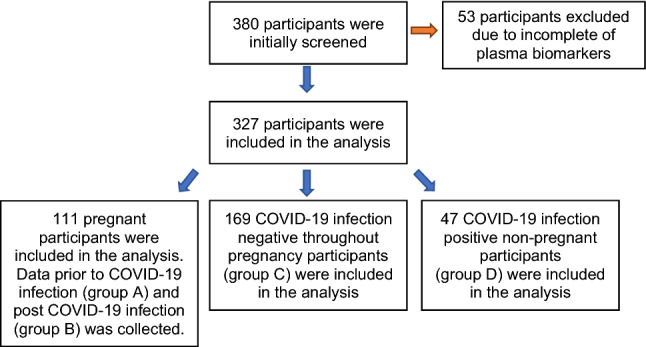
Study participant’s selection process and grouping for analysis

The remaining 327 participants were included in the final analysis and classified into four groups. Group A: COVID-19-negative pregnant females (111 participants); Group B: group A data after COVID-19 infection (111 participants); Group C: COVID-19-negative pregnant females (169 participants) and Group D: COVID-19-positive non-pregnant females (47 participants of reproductive age) (Fig. 1).

Clinical data, laboratory tests, pregnancy outcomes, and foetus outcomes were collected from the hospital’s electronic medical records. Study inclusion criteria were defined as pregnant women who acquired COVID-19 infection during their pregnancy confirmed by a real-time polymerase chain reaction (RT-PCR) (repeated twice) and age-matched non-pregnant COVID-19-positive individuals. Individuals diagnosed with any haematological pathologies defined as anaemia, blood cancers and haemorrhagic conditions or any condition or medications that can suppress bone marrow function were excluded from the study. Results from patients with incomplete medical records were excluded from the final analysis.

The used treatment strategy for the enrolled patients followed the recommended National Health Service (NHS, UK) published COVID-19 management protocols. The study was sponsored by the research and development committee of the Trust (IRAS number 289571) and had ethical approval as a part of our ongoing COVID-19 study of hospital patients. This study was designed and conducted in accordance with the tenets of the Declaration of Helsinki.

### Laboratory procedures

Patients were identified as COVID-19 positive by Reverse Transcriptase Polymerase Chain Reaction (RT-PCR) from throat/nose swabs on a ROCHE COBAS™ analyser (Roche Ltd, Basel, Switzerland). Nasopharyngeal or oropharyngeal samples were collected from patients for the detection of SARS CoV-2 RNA. The Xpert® Xpress SARS-CoV-2 (Cepheid Ltd) real-time RT-PCR assay was performed to achieve qualitative detection of SARS-CoV-2 RNA.

A Sysmex™-XN (Sysmex LTD, Tokyo, Japan) automated haematology analyser was used for routine complete blood count analysis which included haemoglobin, platelets, white blood cells (WBCs), neutrophils, lymphocytes, monocytes, eosinophils, basophils, neutrophils /lymphocytes, neutrophils /eosinophils and finally WBCs* neutrophils /eosinophils termed the CIIR factor. This novel factor was developed to amplify differences within the patient cohort and then observe critical differences between patient groups that may otherwise be lost by analysing biomarker on their own.

### Sample size and statistical analysis

As the study design was multifactorial in nature, it was calculated that a sample size of *n* = 327 is sufficient to provide 95% power at an alpha level of 0.05. All analyses were performed using Statistica® software (version 13.3, StatSoft Inc., Tusla, OK, USA). Distributions of continuous variables were determined by the Shapiro–Wilks test. In cases where the normality of the data could not be confirmed, appropriate data transformations were made, or non-parametric statistical alternatives were used, and categorical variables are expressed as percentages. Univariate associations were determined using Pearson’s (normally distributed data) or Spearman’s method (non-normally distributed data), and forward stepwise regression analyses were performed to test the influence of measured clinical outcomes and the circulatory biomarkers. Differences between groups were subsequently assessed using one-way ANOVA or ANCOVA, as appropriate, followed by Tukey’s post hoc analysis. Comparisons between pregnant and non-pregnant groups were measured using the χ2 or Fisher exact test for categorical variables, whereas the t-test or Mann–Whitney U test was used for continuous variables. p < 0.05 was considered statistically significant.

## Results

### Clinical characteristics

General demographics of the study population are presented in Table 1. There were no significant differences in age, systolic blood pressure, diastolic blood pressure, pulse rate and respiratory rate between all the study groups (all *p* > 0.05). The average age of pregnant patients was 30 years compared with 35.6 years for non-pregnant patients. Of the infected pregnant group, 109 developed mild to moderate respiratory symptoms, and two were admitted to the intensive care unit (ICU). Specifically, one of these patients developed type-1 respiratory failure requiring intubation and extra-corporeal mechanical ventilation (ECMO) and underwent a prolonged recovery period after developing pulmonary fibrosis. She delivered a healthy baby via an emergency caesarean section. The second mother admitted to ICU following a normal vaginal delivery. However, she died of multi-organ failure due to acute fatty liver which was not related to the COVID-19 infection. In both these patients admitted to the ICU, babies survived. Finally, two stillbirths were recorded from the whole cohort of COVID-19 patients. In the first case, documented cause of death was a combination of vascular malperfusion and COVID-19-related placentitis causing premature placental separation. In the second case, there was no documented cause of death; however, foetal and maternal vascular malperfusion may be linked with umbilical cord hypercoiling and stricture as well as prolonged meconium exposure.

**Table 1:** Demographic and clinical observations findings of patients on admission

	Pregnant			Non-pregnant	*p *value
	(Group A) Prior to COVID-19 infection (111 patient)	(Group B) Post COVID-19 infection (111 patient)	(Group C) COVID-19 Negative throughout pregnancy (169 patient)	(Group D) COVID-19-Positive patients (47 patient)	
Age	30 (0.56)	29.42 (0.61)	29.3 (0.45)	35.61 (1.63)	0.80111
SBP	118.55 (15.67)	119.95 (16.50)	119.10 (17.34)	119.06 (16.27)	0.65833
DBP	72.56 (9.63)	75.34 (10.50)	74.65 (8.80)	76.12 (11.20)	0.94454
HR	90.52 (15.44)	92.71 (17.24)	91.85 (19.06)	93.32 (17.24)	0.32467
RR	16.02 (6. 80)	15.90 (6.28)	14.90 (5.88)	16.82 (6.28)	0.87247
O_2_ Saturation	98%	99%	97%	99%	0.21457

Data are presented as mean (standard deviation), *p* values were calculated by a one-way ANOVA or ANCOVA, as appropriate, followed by Tukey’s post hoc analysis. Comparisons between pregnant and non-pregnant groups were measured using the *χ*2 or Fisher exact test for categorical variables, whereas the *t*-test or Mann–Whitney *U* test was used for continuous variables

*SBP* systolic blood pressure, *DBP* diastolic blood pressure, *HR* heart rate, *RR* respiratory rate, *O*_*2*_* saturation* oxygen saturation

^*^Significant *p* values are indicated where *p* < 0.05 was considered significant

### Haematological findings

Peripheral blood analysis showed statistically lower haemoglobin, platelets, lymphocytes, and eosinophils counts in pregnant women post COVID-19 infection compared to their results before acquiring the infection (*p* = 0.00002, *p* = 0.00182, *p* = 0.02672, *p* = 0.00172, respectively) (Table 2). On the other hand, WBCs, neutrophils and monocytes counts were significantly higher in pregnant women after COVID-19 infection than before the infection (*p* = 0.00001, *p* = 0.00001, *p* = 0.00046, respectively). Similarly, other inflammatory blood cell parameters including neutrophils/lymphocytes (NLR) and neutrophils/ eosinophils ratios (NER) were significantly higher (p = 0.00001 and p = 0.00001, respectively) in pregnant women after acquiring the infection compared to their normal pregnancy baseline results. Additionally, the COVID-19-induced immunity response (CIIR) index represented as WBCs × (neutrophils/eosinophils) was higher in COVID-19-positive pregnant women after the infection compared to before infection (*p* = 0.00001). There were no statistically significant differences in any haematological blood cell parameters between pregnant women before the COVID-19 infection and pregnant women who did not obtain the infection during their pregnancy (all *p* > 0.05).

**Table 2:** Haematological findings of the study population

Pregnancy status	Pregnant	Pregnant	*p *value (A vs B)	Pregnant	*p *value (A vs B vs C)	Post hoc analysis	Non-pregnant	*p *value (B vs D)
COVID-19 status	(Group A) Prior to COVID-19 infection	(Group B) Post COVID-19 infection		(Group C) COVID-19 Negative throughout pregnancy			(Group D) COVID-19-Positive patients	
Number of participants	111	111	-	169			47	–
Haemoglobin (115–160 g/L)	122.98 (1.32)	114.40 (1.22)	0.00002*	122.21 (0.98)	0.0001*	A = C > B	119.15 (3.55)	0.8839
Platelets (150–450 × 10^9^/L)	285.67 (6.91)	251.71 (6.43)	0.00182*	282.12 (5.20)	.03144*	A = C > B	273.92 (18.74)	0.8181
WBCs (4–11 × 10^9^/L)	8.72 (0.31)	11.022 (0.29)	0.00001*	8.89 (0.24)	0.001*	A = C < B	5.93 (0.85)	0.00001*
Neutrophil (1.7–7.5 × 10^9^/L)	6.03 (0.28)	8.51 (0.26)	0.00001*	6.04 (0.22)	0.001*	A = C < B	4.008 (0.78)	0.0023*
Lymphocytes (1–4 × 10^9^/L)	1.95 (0.06)	1.72 (0.056)	0.02672*	2.032 (0.045)	0.0349*	A = C > B	1.47 (0.16)	0.62849
Monocytes (0.2*80 × 10^9^/L)	0.57 (0.023)	0.70 (0.02)	0.00046*	0.60 (0.02)	0.0001*	A = C < B	0.38 (0.062)	0.00002*
Eosinophils (> 0.5 × 10^9^/L)	0.15 (0.017)	0.07 (0.015)	0.00172*	0.17 (0.013)	0.004*	A = C > B	0.058 (0.046)	0.9999
Basophils (> 0.1 × 10^9^/L)	0.031 (0.0018)	0.03 (0.002)	0.71904	0.035 (0.001)	0.861	A = B = C	0.018 (0.005)	0.5174
Neut/Lymph	3.32 (0.27)	5.91 (0.25)	0.00001*	3.13 (0.21)	0.0001*	A = C < B	3.90 (0.72)	0.0402*
Neut/Eso	76.65 (15.38)	225.9 (15.71)	0.00001*	84.76 (11.53)	0.0203*	A = C < B	126.72 (49.97)	0.2307
WBCs*Neut/Eos (CIIR)	650.92 (219.91)	2568.95 (224.69)	0.00001*	765.97 (164.88)	0.0001*	A = C < B	802.29 (714.48)	0.0352*

Data are presented as means (standard deviation), *p* values were calculated by one-way ANOVA or ANCOVA, as appropriate, followed by Tukey’s post hoc analysis

*WBCs* white blood cells, *Neut/Lymph* Neutrophil/Lymphocytes, *Neut/Baso* Neutrophil/ Basophils, *Neut/Eso* Neutrophil/ Eosinophils

^*^Significant *p* values are indicated where *p* < 0.05 was considered significant

Non-pregnant COVID-19-positive patients exhibited lower WBCs, neutrophils, monocyte counts and neutrophil/lymphocyte ratio, WBCs*neutrophil/ eosinophil ratios compared to pregnant COVID-19-positive females (*p* = 0.00001, *p* = 0.0023, *p* = 0.00002, *p* = 0.0402, *p* = 0.0352, respectively). Furthermore, no significant difference was found between basophils counts among all the study groups (all *p* > 0.05).

### Delivery and neonatal outcomes

Of the COVID-19-positive group, 73% delivered by vaginal delivery and 27% by caesarean section compared to 66% and 34% in the COVID-19-negative group (*p* = 0.067) (Table 3). Patients who acquired the infection in the third trimester of pregnancy delivered 4.41 (± 1.90) days preterm compared to 24 (± 10.094) days in females who acquired the infection in the second trimester (*p* = 0.044). Indications for early delivery were not reported; however, preterm birth was prevalent in the COVID-19 patients regardless of the severity of the disease. We did not collect these data for COVID-19-negative patients. Data on pregnancy complications from COVID-19 are limited; however, evidence suggests that complications are more common in patients who acquired the infection in the third trimester compared to the second trimester. Among the assessed pregnancies, women affected by COVID-19 in the third trimester showed incidences of maternal haemorrhages (three patients), gestational hypertension (four patients) and preeclampsia (one patient). Pregnancy complications were not observed in the pregnancies affected by COVID-19 infection in the second trimester.

**Table 3:** Delivery and neonate outcomes up to 5 min of birth

	COVID-19-positive pregnancy (111 patients)	COVID-19-negative pregnancy (169 patients)	*p *value
Vaginal delivery	56 (73%)	111 (66%)	
* Spontaneous*	37 (50%)	93 (55%)	
* Induced*	19 (27%)	18 (11%)	0.067
C-Section	18 (27%)	58 (34%)	
Early delivery (days)			–
* 3rd Trimester*	− 4.41 (1.90)		–
* 2nd Trimester*	− 24 (10.094)		–
Birth Weight (g)	2894.37 (67.50)	3194.16 (50.61)	0.02379*
Apgar scale			
* 01*	7.66 (0.21)	8.90 (0.16)	0.00741*
* 05*	8.4 (0.16)	8.88 (0.12)	0.02016*

Data are presented as mean (standard deviation), *p* values were calculated by a *t*-test, (−) represents data not obtained

^*^Significant *p* values are indicated where *p* < 0.05 was considered significant

Pregnant participants in all the groups delivered live-born neonates with two stillborn babies in the COVID-19-positive group. In the 280 born babies, no cases of vertical transmission were reported, and no sets of twins were delivered. The average weight of the babies in the COVID-positive group was significantly lower than the COVID-19-negative group (2894.37 (± 67.50) g compared with 3194.16 (± 50.61) g, *p* = 0.02379) (Table 3).

**Fig. 2:**
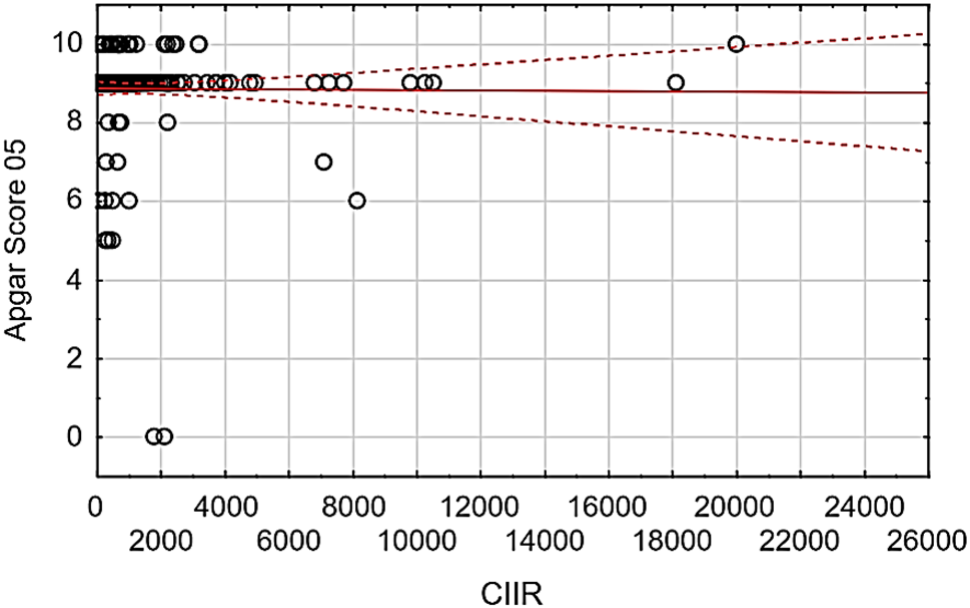
Correlation between the Apgar Score after five minutes and the COVID-19-induced immunity response factor. Univariate associations were determined Spearman’s method (non-normally distributed data), and forward stepwise regression analyses were performed to test the influence of measured clinical outcomes and the circulatory biomarkers

Using the Apgar risk score to evaluate the newborn babies’ health after 1 and 5 min, neonates of COVID-19-negative mothers showed higher Apgar scores compared to that of COVID-19-positive mothers (*p* = 0.00741 and *p* = 0.02016, respectively). Twenty-one babies in the COVID-19-positive group scored six or less in the Apgar risk score. The Apgar risk scores (5 min) were significantly inversely correlated with the COVID-19-induced immunity response factor (*r* = − 0.162, Fig. 2).

## Discussion

The results of this cohort study provide several important insights of the impact of severe acute respiratory syndrome coronavirus-2 (SARS-CoV-2) on pregnancy, the impact of pregnancy on the course of the disease and the implications of the virus on pregnancy outcomes observed from a single centre in the West Midlands, UK. Pregnancy caused an increased immune response to COVID-19; however, infection led to an increase in preterm delivery, a decrease in neonatal birth weight and an increase in neonatal distress defined by the Apgar score. We suggest the use of a novel COVID-19 Induced Immunity Response (CIIR) index as a practical indicator for neonatal distress.

Pregnancy is often considered a partial immunosuppressive state [19]; hence, the interaction of specific pathogens with the foetal/placental unit and the maternal response depending on pregnancy gestation should be studied to optimise prophylaxis or therapy. Similar to previous findings, our study found leucocytosis and lymphopenia are prevalent in COVID-19-positive women compared to non-pregnant and COVID-19-negative individuals along with neutrophilia [4]. However, in addition to this, our study observed a more pronounced immune response in pregnant COVID-19 patients compared with non-pregnant. In more severe cases, we noticed uncontrolled inflammatory and immune responses characterised by high neutrophil/lymphocyte (NLR) and neutrophil/eosinophil (NER) ratios and progression to pregnancy complications such as haemorrhage, hypertension and preeclampsia was positively associated with increased NLR. Furthermore, we tested a novel index termed COVID-19-induced immunity response factor (CIIR, defined as (WBC × neutrophil)/ eosinophil) and compared to non-pregnant COVID-19-positive women, to their pregnant counterparts who showed a higher CIIR. Eosinophil count decreased more significantly than any other white cell fraction indicating its consumption/activity in the fight against the infection whilst the WBCs yield the total power of the immune response against COVID-19 infection. This immune response power, CIIR, was significantly enhanced in pregnant patients to protect the growing embryo. This suggests pregnancy resulted in an increased immune response perhaps to protect the mother. It has previously been suggested that there is an increased emphasis on infection prevention during pregnancy and that maternal immunity strives to decrease inflammatory events so as not to expose the foetus to potentially dangerous inflammatory signals [19].

Despite the increase in immune response observed by the laboratory markers, no significant difference in clinical observations was noted regardless of gestational age. The increased levels of circulating progesterone and oestrogen during pregnancy increase the tidal volume of the lung, arterial partial oxygen saturation, and minute ventilation, which helps the lungs to be more flexible compromising any added stresses [20]. The immunomodulatory properties of the progesterone positively impact many immune pathways, including immune-mediated injuries [21]. Furthermore, during pregnancy, the circulating levels of interleukin-1 receptor antagonist and tumour necrosis factor-α (TNF) receptor increase while the plasma levels of interleukin-1β and TNF‐α decrease, which adds to the physiological protective response against the virus [22]. Adding to this, as it was reported that the COVID-19 virus activates innate and adaptive immune responses, pregnant women could be protected by pregnancy-associated immunomodulation [23]. This further supports our earlier suggestion of pregnancy inducing a hyper-protective state for the mother.

Considering the pro-inflammatory state accompanying pregnancy in the first and third trimesters, this study found higher pregnancy-associated complications in individuals who acquired the infection in the third trimester of pregnancy compared to the second trimester [24]. Nonetheless women gave birth through both vaginal and caesarean section delivery with the percentage of women delivering by caesarean section similar in the COVID-19-positive and -negative females. This indeed reflects the clinical stability of the COVID-19-positive cases with no evidence for any potential respiratory complications during labour to promote an elective caesarean decision. In our study, patients with SARS-CoV-2 had a higher incidence of preterm delivery, especially individuals who acquired the infection in the second trimester. Our results support the observations during early pandemic case series and reports describing preterm deliveries and early ruptures of the membranes in COVID-19-infected women [6, 11, 25]. One suggested explanation of this is the well-known link between the activation of the inflammatory pathways resulting in inflammation of the placenta, termed acute chorioamnionitis and the premature rupture of the membranes and preterm deliveries [26, 27]. In our opinion, the activation of these inflammatory pathways (macrophages and IL-6) in COVID-19 infections and the evidence from other studies highlighting Interleukins and cytokines as markers of preterm delivery in normal pregnancies [28–31] are a case for further evaluation of this association in COVID-19-positive pregnant women.

Our study observed lower birth weights and Apgar scores following COVID-19 maternal infection. Since an increase in preterm delivery was observed in COVID-19-positive females, it is expected to see a reduction in neonatal birth weight. Premature babies are at increased risk of sustaining a range of short and long term complications of prematurity [32, 33]. The Apgar score is not intended for prediction of outcome beyond the immediate postnatal period; however, as low scores correlate with prenatal and perinatal issues, many studies have examined the relationship between low scores (< 7), duration of low scores and subsequent respiratory distress [34], neurologic disability and poor cognitive function [35, 36]. Importantly, the CIIR was inversely correlated with the neonates Apgar risk score five minutes after birth suggesting this marker may be of use in predicting neonates that will require intensive care. Currently, there is growing evidence supporting the COVID-19-induced intrauterine inflammation can cause placental histopathological changes and adverse obstetric and neonatal events, including maternal and foetal vascular malperfusion, infarction, chorioamnionitis and umbilical arteritis [37, 38]. This involvement of the placenta in COVID-19 infection and its consequent complications can explain our findings and support our recommendation to use the (CIIR) ratio as an early indicator of COVID-19-induced maternal and foetal complications.

Overall, in COVID-19-infected pregnant patients, we observed 1.8% were admitted to ICU and 0.9% died, furthermore, 1.8% of neonates died. We did not find COVID-19 listed as a cause of death; however, infection may have precipitated the outcome. A study collating routine clinical data (in select cases, samples for research and development) from a network of over 300 NHS hospitals across the UK between March 2020 and February 2021 observed, of symptomatic pregnant women hospitalised with COVID-19, 10% received critical care and 1% died [39]. Furthermore, findings from a study collating data from UK and USA registries of pregnancies with COVID-19 infection observed the proportions of pregnancies affected by stillbirth was not higher than historical and contemporaneous UK and USA data. They also observed maternal death was uncommon; however, the rate was higher than expected based on UK and USA population data, owing to under ascertainment of patients affected by mild or asymptomatic infection [40]. A systematic review considering neonatal outcomes associated with COVID-19-infected pregnancies found the incidence of preterm births, low birth weight, C-section, neonatal ICU admission appear higher than the general population [41]. Thus, it seems that infection with COVID-19 does not lead to overwhelming maternal complications or increased mortality in the pregnant patient, although admission to ICU is variable. Furthermore, infection could lead to neonatal complications further highlighting the potential benefits of our proposed CIIR score to predict which neonates need intensive care.

Our study has limitations. First, due to the retrospective study design, not all laboratory tests were carried out or recorded in all patients, therefore, their role might be underestimated in predicting in-hospital outcomes. Second, lack of information regarding drug treatment might have also affected the clinical outcomes in some patients. Further, information of any treatment for chronic conditions was not collected in this study and could have affected clinical outcomes. Third, interpretation of our findings might be limited by the sample size. However, by including all pregnant COVID-19-positive patient across the trust, we believe our study population is representative of cases diagnosed and treated in West Birmingham. Finally, serological data, asymptomatic or overlooked COVID-19 subjects were missing from the study and we did not observe any pregnant women infected with COVID-19 in the first trimester.

SARS-CoV-2 is a complex disease; understanding its impact on both the mother and the foetus is crucial to protect both from adverse effects. Our study describes a range of haematological blood parameters and clinical findings that can help early detection of patients at risk of developing maternal and foetal complications. Timely reporting of pregnancy status, exposure time, symptoms, clinical presentation, and laboratory abnormalities are critical in developing appropriate evaluation and management plans for pregnant patients with COVID-19 infection. The authors of this study would recommend routine evaluation of the inflammatory blood cell parameters and CIIR ratio in assessing COVID-19-positive pregnant patients to help predict maternal and neonatal complications. These data can help improve the management of COVID-19-infected pregnant patients and their neonates whilst building clinical guidance for treating COVID-19 during pregnancy.

## Data Availability

The data that support the findings of this study are available on request from the corresponding author.
